# Efficacy of *Artemisia annua* L. extract for recovery of acute liver failure

**DOI:** 10.1002/fsn3.1662

**Published:** 2020-06-05

**Authors:** Chan Young Park, Eunyong Choi, Hee‐Jin Yang, Seong Hyun Ho, Su‐Jin Park, Ki‐Moon Park, Seon‐Hee Kim

**Affiliations:** ^1^ Sungkyun Biotech. Co. LTD. Suwon Korea; ^2^ G&P Bioscience Co. LTD. Goyang Korea; ^3^ Department of Food Science & Biotechnology Sungkyunkwan University Suwon Korea

**Keywords:** *Artemisia annua* L., inflammation, liver failure, NF‐κB translocation, oxidative stress

## Abstract

*Artemisia annua* L. is an annual herb belonging to the Asteraceae family. It is commonly grown in parts of Asia, including Korea and China, and is called by its nickname Gae‐ddong‐ssuk, or Chung‐ho. The herb is well known for its positive effects on fever and hemostasis, as well as its antibiotic effects. To evaluate the protective properties of *A. annua* L. on the liver, an acute liver failure animal model was set up with intraperitoneal injection of lipopolysaccharide (LPS) and D‐galactosamine (D‐galN) in C57BL/6J mice, showing increased levels of AST (aspartate transaminase) and ALT (alanine transaminase). Oral administration of the extract of *A. annua* L. (EAA) for 2 weeks reduced the level of AST and ALT up to 50% of the levels in the negative control group treated with water vehicle. The efficacy of EAA was more effective than that in a comparative positive control group treated with milk thistle extract. Moreover, EAA protected hepatic cells and tissues from oxidative stresses and inflammatory damages, showing downregulation of inflammatory cytokines such as interleukin‐1 beta (IL‐1β), interleukin‐6 (IL‐6), and tumor necrosis factor‐alpha (TNF‐α). We also found that LPS stimulated the mouse macrophage cell line, Raw264.7, and secreted a tremendous level of proinflammatory cytokines and the secretion of these cytokines was reduced with EAA treatment via downregulation of mitogen‐activated protein kinase phosphorylation and p65 translocation. This study demonstrated that *A. annua* L. extract is a promising treatment for protection against and recovery from liver damage, as well as maintenance of liver health.

## Graphical abstract

This study investigated the efficiency of *Artemisia annua* L. for protection against and treatment of liver damage using a liver failure mice model. Mice were administered *A. annua* L. extract (EAA) for 2 weeks, and levels of AST (aspartate transaminase) and ALT (alanine transaminase) were significantly decreased compared to mice treated with milk thistle extract (controls). EAA protected hepatic cells and tissues from oxidative stresses and inflammatory damages by downregulating inflammatory cytokines such as interleukin‐1 beta (IL‐1β), interleukin‐6 (IL‐6), and tumor necrosis factor‐alpha (TNF‐α).

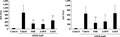

## 1 INTRODUCTION

The liver is an organ that plays a pivotal role in recognizing various toxic substances from the outside and discharging them to the outside of the body. It is known that nonalcoholic fatty liver disease (NAFLD) is reported in 20%–30% of normal adults who do not have any particular liver disease (Bellentani, Scaglioni, Marino, & Bedogni, 2010; Younossi et al., 2011). The prevalence of NAFLD in Korean adults is reported to be about 16%–50% (Lee et al., 2007; Park et al., 2006). In general, the prevalence of NAFLD is higher in obese patients (Angulo, 2002; Wanless & Lentz, 1990). It is believed that insulin resistance that occurs as a result of obesity is one of the major causes of lipid deposition in the liver. Unlike simple steatosis, nonalcoholic steatohepatitis (NASH) is associated with pathological findings such as ballooning degeneration, cell death, and inflammatory infiltration (Cohen, Horton, & Hobbs, 2011). The prevalence of NASH is about 2%–5% worldwide (Bellentani et al., 2010) and about 2% in Korea (Lee et al., 2007). It is reported that 10%–29% of patients with NASH develop cirrhosis within 10 years, and 4%–27% of patients with cirrhosis develop liver cancer (Argo & Caldwell, 2009; Starley, Calcagno, & Harrison, 2010). Damage to liver function and liver disease are difficult to recover from and often fatal.

Acute liver injury caused by hepatotoxic drugs is mainly associated with gram‐negative bacterial endotoxin (Bower, Johns, Margolis, Williams, & Bell, 2007). Lipopolysaccharide (LPS), a major component of gram‐negative bacteria, plays an important role in the initiation phase of endotoxic damage and activates inflammatory cytokines that cause liver tissue damage. D‐galactosamine (D‐GalN), a liver‐specific toxin, increases the sensitivity of endotoxins such as LPS, induces liver toxicity within a few hours, selectively depletes nucleotides liberated from hepatocytes, and suppresses protein synthesis (Galanos, Freudenberg, & Reutter, 1979). Therefore, the LPS and D‐GalN acute liver injury model is widely used for the development of the pathogenesis of liver injury and drug development, and this model induces typical hepatocyte necrosis and apoptosis (Eipel et al., 2007).

Increased reactive oxygen species (ROS) by LPS/D‐GalN treatment activates macrophages in liver tissues and induces tumor necrosis factor‐alpha (TNF‐α), interleukin‐6 (IL‐6), and interleukin‐1 (IL‐1) (Mayer & Spitzer, 1993; Neihorster, Inoue, & Wendel, 1992). Inflammatory cytokines induce hepatocyte necrosis and decrease the activity of antioxidant enzymes (Yang et al., 2014). Cycloxygenase‐2 (COX2) induced by inflammatory stimuli produces prostaglandin E2 (prostaglandin E2, PGE2), an inflammation‐promoting substance (Jung et al., 2013), and TNF‐α is involved in apoptosis (Streetz et al., 2000). Thus, inhibition of reactive oxygen and inflammatory cytokines can be an important strategy for the prevention and treatment of acute liver injury by LPS/ D‐GalN.

Lipopolysaccharide associates and activates Toll‐like receptor (TLR)4 on the cell surface. The TLR4 signal initiates translocation from the cytoplasm to the nucleus of the proinflammatory transcription factor nuclear factor‐kappa light chain enhancer (NF‐κB) of activated B cells and induces gene transcription (Kawai & Akira, 2010; Ni et al., 2020). In the nucleus, NF‐κB is inactivated and released from the nucleus by a newly synthesized protein‐inhibiting κB (IκB) (Nelson et al., 2004; Wang et al., 2020). The dynamics of NF‐κB translocations in and around the nucleus are thought to contribute to the expression of inflammatory genes (Ashall et al., 2009; Nelson et al., 2004). In addition, LPS facilitates mitogen‐activated protein kinases (MAPKs) such as JUN n‐terminal kinase (JNK) and transcriptional factor, nuclear factor‐kappa B (NF‐κB), leading to the secretion of cytokines. Therefore, LPS is used widely as an experimental model for studying inflammatory response, and damage to various tissues has been tested on animals.

D‐galactosamine (D‐GalN), a family of aminosulfate, induces toxicity in hepatocytes and causes hepatocyte fat changes, inflammatory cell infiltration, and hepatocyte necrosis in the treated animals (Farber, Gill, & Konishi, 1973; Keppler, Lesch, Reutter, & Decker, 1968). In addition, D‐GalN is known to cause hepatic damage by inhibiting mRNA synthesis and protein synthesis through galactose metabolism in the liver (Wang & Wendel, 1990; Xu et al., 2019). D‐GalN is also reported to alter the permeability of the mucosa, thereby increasing the uptake of endotoxin, which interferes with the repair process of cell membranes, leading to hepatotoxicity (Wang, Wang, Li, Han, & Gong, 2013). Administration of D‐galN, together with bacterial LPS, has been extensively studied, both biochemically and histologically, as an experimental model of hepatitis, as it causes liver damage similar to human hepatitis (Wu et al., 2014).

Considering the severity of liver disease and the lack of therapeutic agents, the discovery of functional food materials with preventive effects related to liver disease is necessary. *Artemisia annua* is a plant native to East Asia, particularly Korea, China, and Mongolia (Räth et al., 2004). Antimalarial artemisinin has been isolated from its extract, and it has become known worldwide (Tu, 2011). Artemisinin and its derivatives have been reported as effective in the treatment of pathogenic diseases such as viruses, bacteria, and fungi, in addition to the therapeutic effects of malaria (Birku, Mekonnen, Bjorkman, & Wolday, 2002; Ding, Beck, & Raso, 2011; Ho, Peh, Chan, & Wong, 2014). Recently, artemisinin and its derivatives have been tested for treatment of respiratory diseases such as asthma and treatment of various cancers through strong anti‐inflammatory reactions (Efferth, Dunstan, Sauerbrey, Miyachi, & Chitambar, 2001; Ho, Alcazar, Fujii, Hirshman, & Goodyear, 2004). Not only the compounds isolated from *A. annua*, but also its extract have been reported to have antiobesity and anticancer effects (Kim et al., 2017). In particular, clinical trials of the extracts have been conducted for patients with rheumatoid arthritis and degenerative osteoarthritis (Hunt, Stebbings, & McNamara, 2016; Yang et al., 2017).

This study aimed to demonstrate that *A. annua* L. extract is a promising treatment for protection against and recovery from liver damage, as well as maintenance of liver health.

## 2 MATERIALS AND METHODS

### 2.1 Plant extract

*Artemisia annua* L. was purchased commercially in Korea. *Artemisia annua* L. was extracted with water (labeled AAWE) and 25% ethanol (labeled AAEE) in an extractor at 85°C for 4 hr. These extracts were filtered with filter paper and used for in vitro experiments. For the other functional assays, the extracts were filtered with 10‐µm cartilage filter and the filtrates were concentrated to 25% of solid contents under 60°C reduced pressure. The concentrates were mixed with maltodextrin (dextrin 30%: solid content 70% of weight ratio) and powdered using a spray dryer.

### 2.2 Animals and experimental group

C57BL/6 mice (male, 18–23 g; 6 weeks old) were obtained from OrientBio Co., Ltd. and housed in a temperature‐controlled room at 21 ± 2°C and 50 ± 20% humidity with a 12‐hr light/dark cycle. Mice had free access to standard laboratory food and water ad libitum. Before the experiments, they were acclimatized for 1 week. All animal experiments were approved by the Animal Research Ethics Committee of KPC (Approval Number: P173010; Gwangju, Gyeonggi‐do, Korea). Lipopolysaccharide (LPS; *Escherichia coli*, 055: B5) and D‐galactosamine (D‐galN) were purchased from Sigma‐Aldrich.

Fifty mice were divided into five groups: (a) untreated naïve control group, (b) LPS/D‐galN‐treated liver failure group, (c) LPS/D‐galN + SME: *Silybum marianum* extract (100 mg/ml b.w.t) group, (d) LPS/D‐galN + AAWE (100 mg/ml b.w.t) group, and (e) LPS/D‐galN + AAEE (100 mg/ml b.w.t) group. Test materials were administrated orally once a day for 14 days. After 1 hr of the last oral administration, LPS (4 μg/kg) and D‐galN (400 mg/kg) were administrated by intraperitoneal injection. Blood, liver tissues, and spleen tissues were collected for biochemical and histological analyses after LPS/D‐galN treatment for 4 hr.

### 2.3 Measurement of aspartate transaminase (AST) and alanine transaminase (ALT)

The mice were bled for biochemical analysis. The collected blood was centrifuged at 3,000 *g* for 10 min to separate serum. Total levels of AST and ALT were measured using a serum biochemistry analyzer (Accute TBA‐40FR, Toshiba Medical System Co.).

### 2.4 Histological analysis

The liver tissues were fixed in 10% neutral buffered formalin and embedded in paraffin followed slicing into 4‐μm‐thick sections. The tissue sections were stained with hematoxylin and eosin (H&E) and observed under a light microscope (Olympus MVX10 microscope).

### 2.5 Real‐time polymerase chain reaction (PCR)

Total RNA was extracted from liver tissues using NucleoZOL (MACHEREY‐NAGEL Gmbh & Co., KG). The experimental procedure was followed according to manufacturer's protocols. cDNA was synthesized from 0.5 μg of total RNA using a ReverTra Ace qPCR RT Master Mix kit (FSQ‐201; TOYOBO) with random primers. The synthesized cDNA was mixed with the amplification mixture containing THUNDERBIRD™1 SYBR^Ⓡ^ 29 qPCR Mix (QPS‐201; TOYOBO) and primers. cDNA was subjected to 40 amplification cycles of PCR using a Thermal Cycler Dice (Takara) normalized with 36B4 expression. Oligonucleotides used for PCR are listed in Table 1.

**TABLE 1:** Polymerase chain reaction primers used in this study

Primer	Direction	Sequence
36B4	Forward	AGATGCAGCAGATCCGCAT
36B4	Reverse	GTTCTTGCCCATCAGCACC
TNF‐α	Forward	AAGCCTGTAGCCCACGTCGTA
TNF‐α	Reverse	GGCACCACTAGTTGGTTGTCTTTG
IL‐1β	Forward	TCCAGGATGAGGACATGAGCAC
IL‐1β	Reverse	GAACGTCACACACCAGCAGGTTA
IL‐6	Forward	CCACTTCACAAGTCGGAGGCTTA
IL‐6	Reverse	GCAAGTGCATCATCGTTGTTCATA
TLR4	Forward	ACCTGGCTGGTTTACACGTC
TLR4	Reverse	ACACCTGCCAGAGACATTGC

Abbreviations: IL‐1β, interleukin‐1‐beta; IL‐6, interleukin‐6; TLR‐4, Toll‐like receptor‐4; TNF‐α, tumor necrosis factor‐alpha.

### 2.6 Cell culture

The murine macrophage RAW264.7 cell line was purchased from the American Type Culture Collection (ATCC). The cells were cultured in Dulbecco modified Eagle medium (DMEM) (HyClone; GE Healthcare Life Sciences) containing 10% fetal bovine serum (HyClone FBS; GE Healthcare Life Sciences) and 1% (v/v) penicillin–streptomycin solution (HyClone Penicillin–Streptomycin solution; GE Healthcare Life Sciences). Cells were maintained at 37°C in a 5% CO_2_ incubator.

### 2.7 Viability assay

For the cell viability test, RAW264.7 cells were seeded into 96‐well plates at a density of 1 × 10^5^ cells/ml and cultured in DMEM for 24 hr. After cells were attached, various concentrations of *A. annua* water extract (AAWE) was applied to each well and cultured for another 24 hr. Medium was replaced with fresh medium containing 0.5 mg/ml thiazolyl blue tetrazolium bromide (MTT) (AppliChem), and plates were incubated for 4 hr in a dark condition at 37°C. After medium was removed, insoluble formazan was dissolved with 100 μl of dimethyl sulfoxide and shaken for 30 min. The optical absorbance of the dissolved sample was measured at 540 nm with an ELISA plate reader (SpectraMax M2/M2e Microplate Readers; Molecular Devices).

### 2.8 Nitric oxide (NO) assay and cytokine assay

One hundred thousand cells/ml of RAW264.7 was cultured in DMEM media without phenol red (HyClone; GE Healthcare Life Sciences) in 24‐well plates for 24 hr. After then, cells were exposed to various concentration of *A. annua* water extract (AAWE) with or without 100 ng/ml LPS for another 24 hr. Each medium was collected and centrifuged for 10 min at 25,000 *g* to obtain clean supernatants. These supernatants were used in the NO assay and cytokine assay.

For measuring the NO contents, Griess reagent I (0.2% 1,2‐di‐1‐naphthyl‐ethylenediamine dihydrochloride) and Griess reagent II (2% sulfanilamide in 10% phosphoric acid) were made manually and mixed to a 1:1 ratio. The same amount of culture medium samples and Griess reagent mixture was mixed together for 10 min at room temperature in the dark condition. Using an ELISA plate reader, the absorbance was measured at 540 nm. NO contents were calculated on the basis of sodium nitrite standard curves.

For measuring the contents of mouse TNF‐α and IL‐6, commercial enzyme‐linked immunosorbent assay (ELISA) kits were used and the procedures followed the manufacturer (R&D Systems) instructions. The optical density of each well was determined using a microplate reader at 450 nm.

### 2.9 Western blot analysis

Raw264.6 cells were seeded at a density of 1 × 10^5^ cells/ml in a 100‐mm culture dish and grown to 90% confluence. Various concentrations of *A. annua* water extract (AAWE) were applied to the cells with or without 100 ng/ml LPS for 24 hr. After 24 hr, cells were harvested and washed with PBS to get clean cell pellets. For protein isolation of cytoplasmic and nuclear fraction, a commercial nuclear and cytoplasmic extraction kit was used (NE‐PER™ Nuclear and Cytoplasmic Extraction Reagents; Thermo Scientific). The protein isolation procedure was performed according to the manufacturer's protocols. Total protein was extracted using a RIPA buffer supplemented with protease and phosphatase inhibitor, and the protein was quantified based on the Bradford dye‐binding method (Bio‐Rad). The electrophoresed protein on 10%–15% SDS‐PAGE was transferred to nitrocellulose membranes (GE Healthcare). The probed membranes were blocked with 5% BSA solution for 1 hr at 4°C and incubated with primary antibody overnight at 4°C. Primary antibodies we used were as follows: NF‐κB p65 (1:1,000, #8242), Phospho‐p38 MAPK (1:1,000, #9211), p38 MAPK (1:1,000, #9212), Phospho‐p44/42 MAPK (1:1,000, #9101), p44/42 MAPK (1:1,000, #9102), Phospho‐SAPK/JNK (1:1,000, #9251), SAPK/JNK (1:1,000, #9252), and Histone H3 (1:1,000, #9715). All products were purchased from Cell Signaling Technologies. Membranes were washed three times with 1xTBST solution, and secondary antibody dissolved in 5% skim milk was probed at room temperature for 2 hr. Secondary antibodies used were as follows: anti‐rabbit IgG (1:5,000, #7074) from Cell Signaling and β‐actin (1:5,000, sc‐47778) from Santa Cruz Biotechnology. Membranes were washed five times, and protein activity was detected using ECL (Amersham ECL Prime Western Blotting Detection Reagent; GE Healthcare). Quantification of protein expression was calculated using ImageJ.

### 2.10 Statistical analysis

Data are expressed as means ± standard deviation (*SD*) and tested using SPSS (version 20, IBM SPSS Statistics). The differences between groups were assessed using one‐way analysis of variance (ANOVA) followed by Fisher's least significant difference (LSD) test and Dunnett's T3 post hoc test, with *p* < .05 and *p* < .001 considered statistically significant, respectively.

## 3 RESULTS

### 3.1 AAWE decreased levels of blood AST and ALT from liver failure mice

**FIGURE 1:**
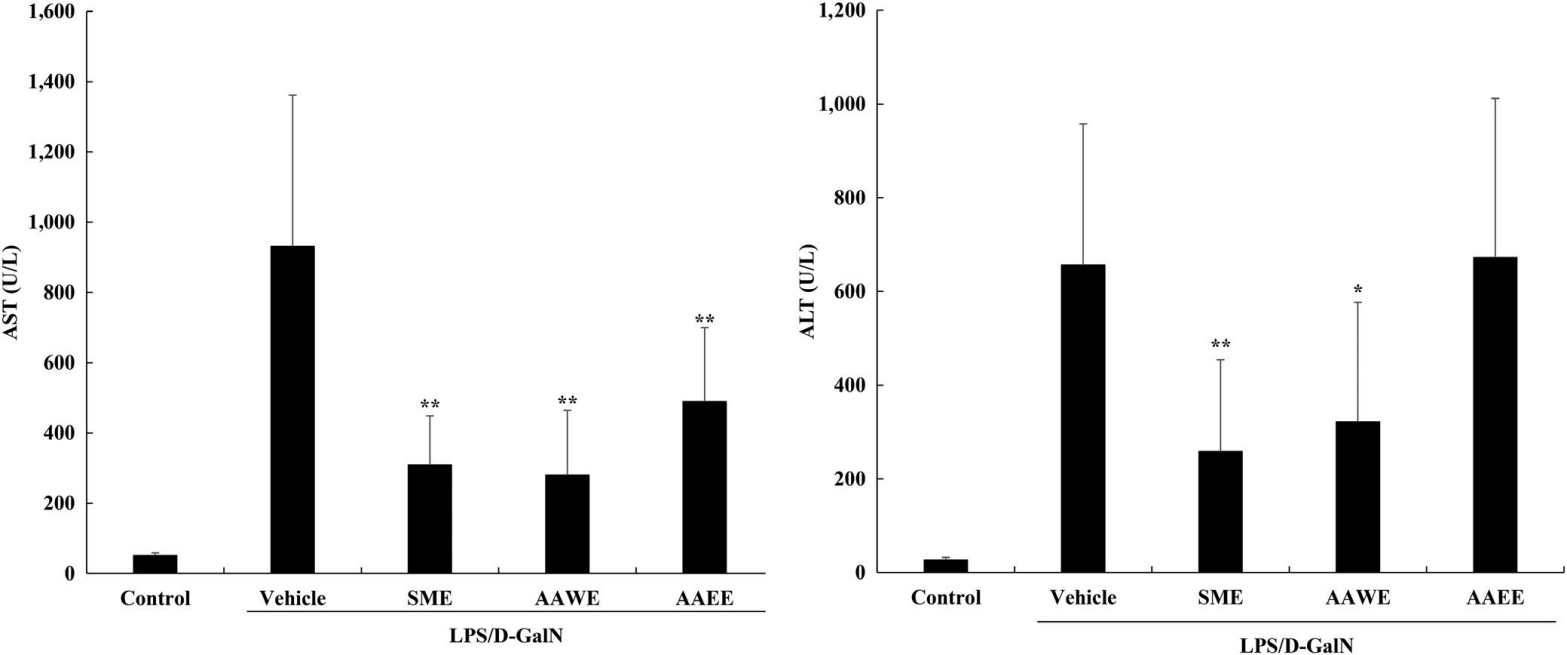
Effects of *Artemisia annua e*xtract on aspartate aminotransferase (AST) and alanine amino aminotransferase (ALT) levels in C57BL/6 mice after lipopolysaccharide/D‐galactosamine (LPS/D‐GalN) treatment. Data are expressed as mean ± *SD* (*n* = 10 per group). Significantly different from Vehicle group, *p < .05 (vs. vehicle group); **p < .01 (vs. vehicle group). Mice are administered orally with test materials once a day for 14 days before LPS 4 μg/kg, D‐GalN 400 mg/kg administration. Serum is collected 3 hr after the administration, and plasma transaminase activity is determined. AAEE, *Artemisia annua* 25% ethanol extract 100 mg/kg; AAWE, *Artemisia annua* water extract 100 mg/kg; SME, *Silybum marianum* extract 100 mg/kg

Mice were fed with test materials (drinking water, SME, AAWE, or AAEE) once a day for 14 days. LPS (4 μg/kg) and D‐galN (400 mg/kg) were injected intraperitoneally 4 hr prior to sacrifice on the last day. Intraperitoneal injection of LPS and D‐GalN induced liver toxicity within a few hours, selectively depleting the nucleotides liberated from hepatocytes. As the hepatocyte necrosis and hepatic tissue destruction progresses due to hepatotoxicity, transaminases are liberated into the blood and exhibit high activity. AST activity in the blood from untreated naïve animals (negative control group) was found to be 50.60 ± 8.17 U/L, while the level of AST increased dramatically to 930.67 ± 430.71 U/L in the LPS/D‐galN‐treated animals (vehicle group). Oral administration of *A. annua* extract (AAWE or AAEE) significantly decreased the blood AST levels in the LPS/D‐galN‐treated animals to 280.40 ± 184.53 and 488.90 ± 211.27 U/L, respectively (Figure 1a). Compared with the active comparison group (SME: *Silybum marianum* extract, which is known for liver function improvement efficacy), the level of AST was lower with AAWE than the 308.60 ± 140.00 U/L seen with SME.

LPS/D‐galN treatment also increased the level of blood ALT from the normal base line (27.00 ± 5.31 U/L up to 656.44 ± 301.71 U/L) (Figure 1b). Unlike AST, the ethanol extract of *A. annua* was not able to change the increased level of ALT. However, the water extract of *A. annua* (AAWE) showed significant reduction in blood ALT at a level of 321.10 ± 255.72 U/L (*p* < .05). The level of AAWE was comparable to the ALT level with SME (258.60 ± 195.15 U/L). Administration of SME and AAWE demonstrated suppression of increased ALT activity by 51.08% and 60.61%, respectively.

### 3.2 AAWE enhanced survival rates of liver failure mice damaged with a lethal dose of LPS/G‐galN

**FIGURE 2:**
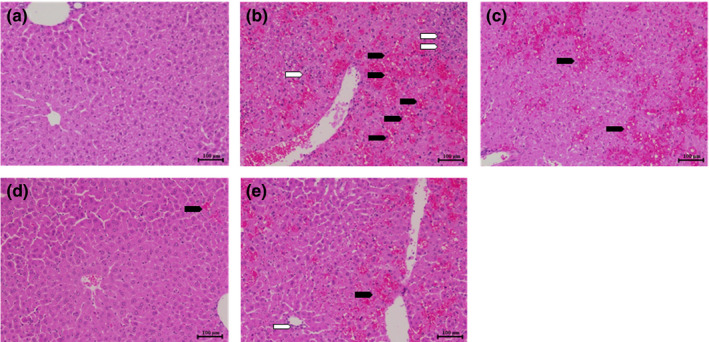
Protective effects of *Artemisia annua* extract on liver injury in C57BL/6 mice after lipopolysaccharide/D‐galactosamine (LPS/D‐GalN) treatment. Mice are administered orally with test materials once a day for 14 days before LPS 4 μg/kg, D‐GalN 400 mg/kg administration. Histologic examination of the liver in mice 3 hr after LPS/D‐GalN injection with distilled water, infiltration of inflammatory cells (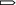), and massive necrosis associated with intralobular hemorrhage (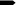). Tissues are fixed in 10% formalin, embedded in paraffin, sectioned at 4–5 µm, and stained with hematoxylin and eosin. Magnification = 100×; Scale bar = 200 µm. (a): Control, (b): vehicle control, (c): *Silybum marianum* extract 100 mg/kg, (d): *A. annua* water extract 100 mg/kg, (e): *A. annua* 25% ethanol extract 100 mg/kg

When a lethal dose of LPS/D‐galN (4 μg/kg and 400 mg/kg, respectively) was administrated intraperitoneally, seven out of 10 mice died within 3 hr after injection (Table 2). However, AAWE‐ or AAEE‐treated group showed increased survival rates up to 90% after the same dose of LPS/D‐galN injection. The animals ingested 100 mg/kg of oral AAWE or AAEE for 2 weeks prior to the drug injection. Intake of AAWE or AAEE can effectively protect liver cells from drug damage and protect individuals from fatal acute liver damage. As can be seen in Figure 2, LPS 4 μg/kg and D‐GalN 400 mg/kg (Figure 2b) injured the hepatocytes and tissues of the animal, causing intravascular bleeding and infiltration of inflammatory cells. In contrast, hepatic bleeding was significantly reduced in the liver tissues of animals that had been treated with AAWE (Figure 2d) or AAEE (Figure 2e) for 2 weeks.

**TABLE 2:** Effects of *Artemisia annua* extract on survival rates in C57BL/6 mice after lipopolysaccharide/D‐galactosamine (LPS/D‐GalN) treatment

	Control	Vehicle	SME	AAWE	AAEE
*n*	10	10	10	10	10
Death	0	7	4	1	1
Survival rate (%)	100	30	60	90	90

Note

Mice were administered orally with test material once a day for 14 days before LPS 4 μg/kg, D‐GalN 400 mg/kg administration. Survival rate was confirmed after challenge.

Abbreviations: AAEE, *Artemisia annua* 25% ethanol extract 100 mg/kg; AAWE, *Artemisia annua* water extract 100 mg/kg; SME, *Silybum marianum* extract 100 mg/kg.

### 3.3 AAWE reduced production of inflammatory cytokines from the damaged liver tissues

**FIGURE 3:**
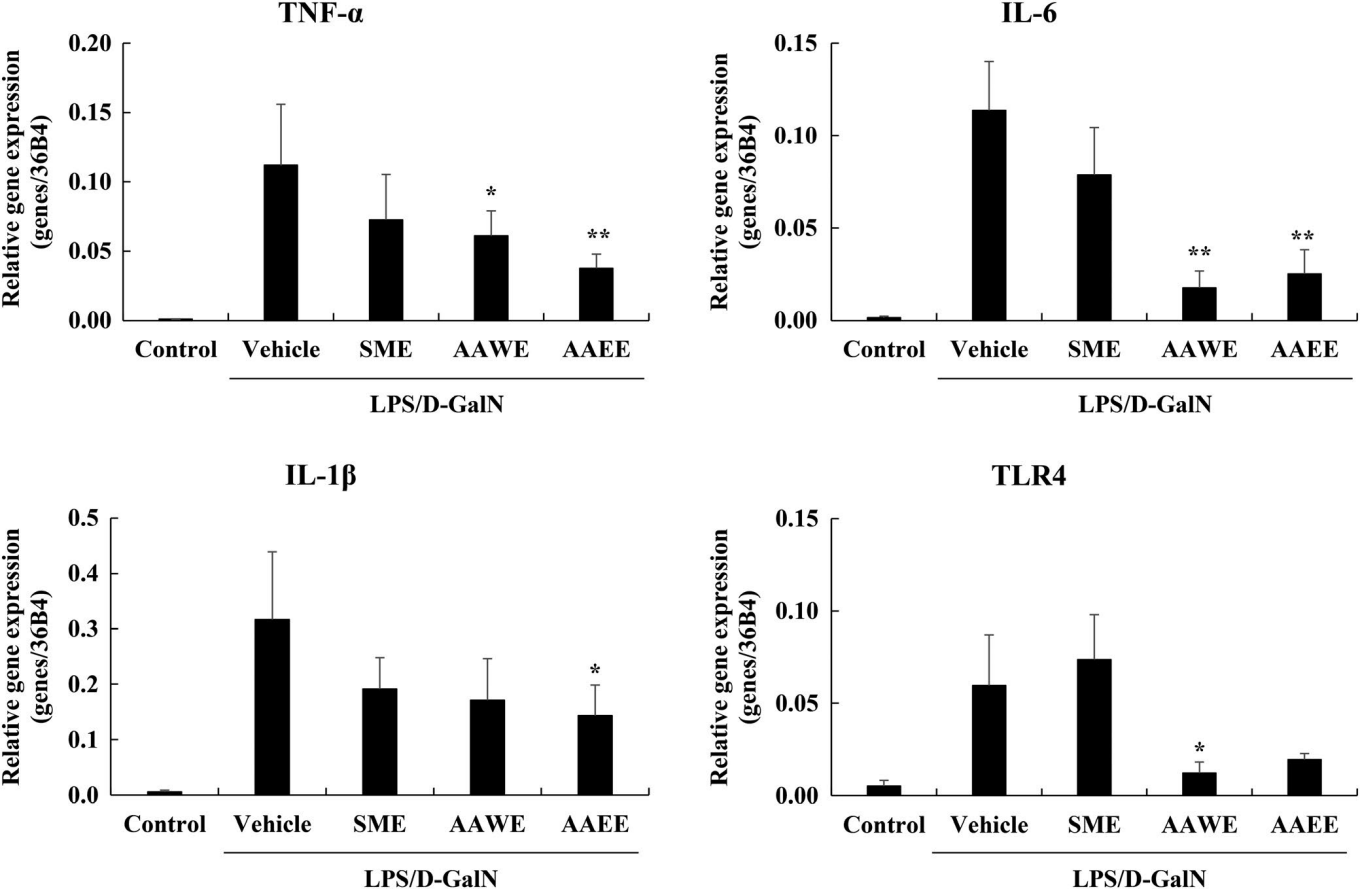
Effects of *Artemisia annua e*xtract on TNF‐α, IL‐1β, IL‐6, and TLR‐4 production in C57BL/6 mice liver tissue after lipopolysaccharide/D‐galactosamine (LPS/D‐GalN) treatment. Data are expressed as mean ± *SD* (*n* = 5 per group). Mice were administered orally with test materials once a day for 14 days before LPS 4 μg/kg, D‐GalN 400 mg/kg administration. Liver tissue is collected 3 hr after LPS/D‐GalN treatment, and mRNA expression of TNF‐α, IL‐1β, IL‐6, and TLR‐4 is measured by real‐time PCR. *p < .05 (vs. vehicle group); **p < .01 (vs. vehicle group). AAEE, *Artemisia annua* 25% ethanol extract 100 mg/kg; AAWE, *Artemisia annua* water extract 100 mg/kg; PCR, polymerase chain reaction; SME, *Silybum marianum* extract 100 mg/kg

After injection of LPS 4 μg/kg and D‐GalN 400 mg/kg, liver tissues from the animals were harvested to examine gene expression related to inflammatory signaling and inflammation. Administration of LPS/D‐galN caused damage to liver tissue and thereby significantly increased the expression of inflammatory precursors TNF‐α, IL‐1β, and IL‐6. In addition, LPS and D‐galN administration increased the expression of Toll‐like receptors (TLRs), which are present on the surface of macrophages and recognize antigenic molecule patterns resulting in triggering of intracellular signaling. The increased expression of TLRs stimulates the active expression of inflammatory cytokines and costimulatory molecules and induces a strong inflammatory response in tissues. In particular, the expression of TLR4, which recognizes LPS as a ligand, was increased in liver tissues (Figure 3). In liver tissues of the animals fed AAWE 2 weeks prior to LPS and D‐galN administration, TLR4 expression was significantly decreased compared to the livers of animals treated with vehicles only after LPS and D‐galN injection. This showed significantly better regulation in the early stages of inflammation than the group that received SME, *S. marianum* extract, one of the active substances we compared. The decrease in TLR4 expression by AAWE will be the starting point to sequentially reduce the expression of other inflammatory cytokines and inflammatory precursors. AAWE reduced TNF‐α expression by 54.5% compared to the vehicle group and decreased 15.5% of IL‐6 and 54.0% of IL‐1β in a similar manner. There was no difference in the suppression effect on the expression of inflammatory precursors between the AAWE and AAEE groups. These *A. annua* extracts demonstrated a stronger anti‐inflammatory effect compared to SME. The liver tissue from animals that received AAWE expressed much less of the TLR4 gene compared to the tissues from animals that received SME, and similar reductions in production of TNF‐α and IL‐6 (84.3% and 22.4%, respectively).

### 3.4 AAWE reduced production of inflammatory cytokines in the LPS‐treated immune cells

**FIGURE 4:**
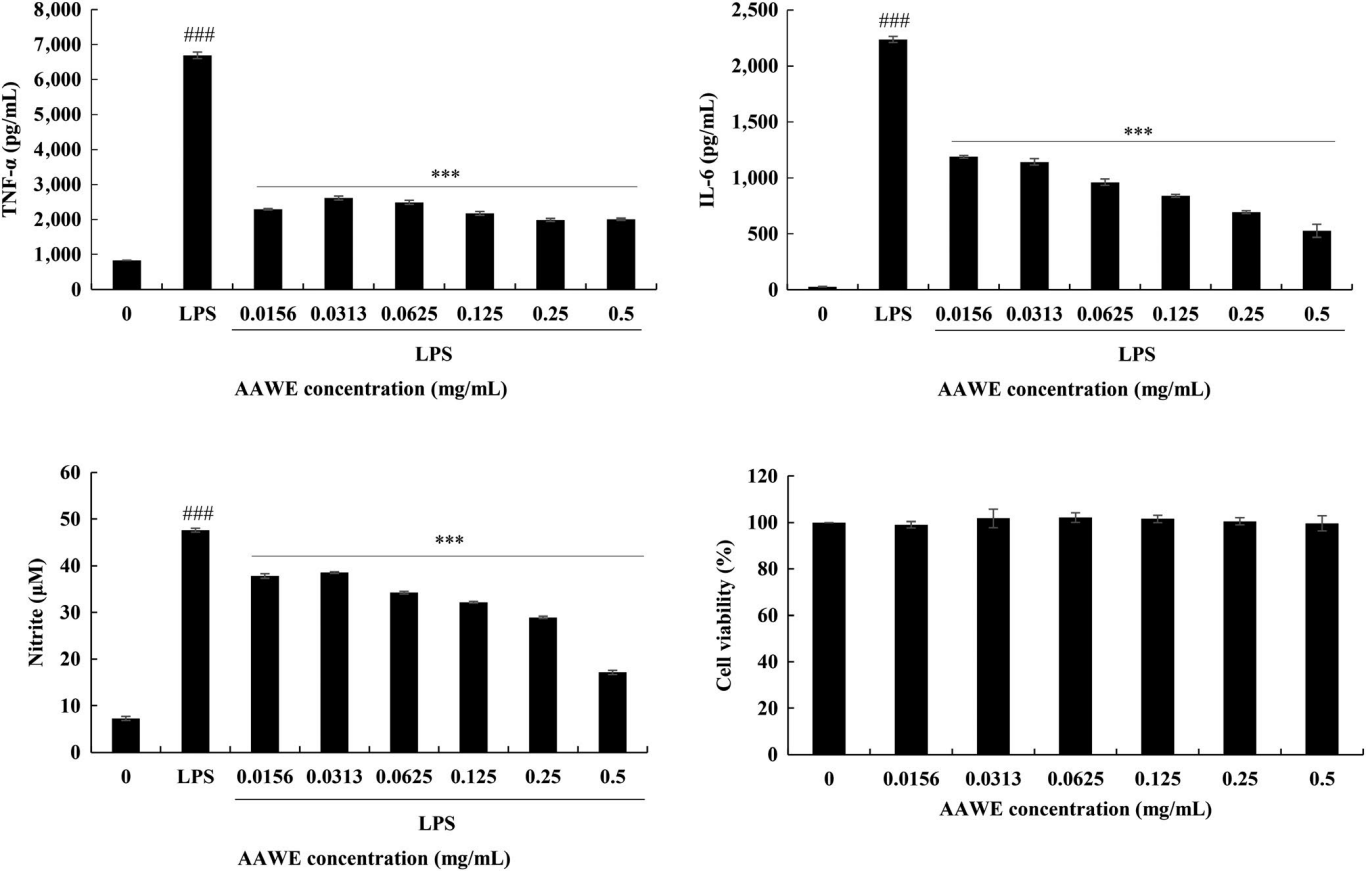
Effect of *Artemisia annua* water extract (AAWE) on TNF‐α, IL‐6, and NO production induced by lipopolysaccharide (LPS) in RAW264.7 cells. Data are expressed as mean ± *SD* of triplicate experiments. Cells are grown for 24 hr and treated with various concentrations of *A. annua* water extract (AAWE) with or without 100 ng/ml of LPS for another 24 hr. Medium is collected for TNF‐α, IL‐6, and NO contents, and cell viability is based on the MTT assay. ***p < .005 (vs. LPS group); ^###^ *p* < .005 (vs. control group). IL‐6, interleukin‐6; MTT, thiazolyl blue tetrazolium bromide; NO, nitrous oxide; *SD*, standard deviation; TNF‐α, tumor necrosis factor‐alpha

Inhibition of the expression of inflammatory precursors and inflammatory cytokines in liver tissues of animals treated with AAWE was the same in the mouse macrophage cell line, Raw264.7 cells (Figure 4). The production of inflammatory cytokines, TNF‐α and IL‐6, increased rapidly to 6,693 and 2,237 pg/ml, respectively, in Raw264.7 stimulated by 100 ng/ml of LPS. TNF‐α production was decreased to a 34% level with a low concentration of SME (0.0156 mg/ml), and a similar reduction was seen with a high concentration (down to 17% with 0.5 mg/ml). In a similar manner, LPS stimulation increased production of NO, the inflammatory precursor to 47.62 μM. When AAWE was exposed to LPS‐stimulated cells with low concentrations of 0.0156 mg/ml to high concentrations of 0.5 mg/ml, the production of IL‐6 and NO decreased with the concentration of the extracts. IL‐6 production was reduced by 2% at a high dose of AAWE, and NO production was also decreased to 13% of the non‐AAWE‐treated cells. It was found that the *A. annua* extract can effectively regulate the inflammatory response caused by cell damage and protect the cells against the invasion of extreme foreign substances such as LPS stimulation.

### 3.5 AAWE reduced NF‐κB translocation into the nucleus in the LPS‐treated immune cells

**FIGURE 5:**
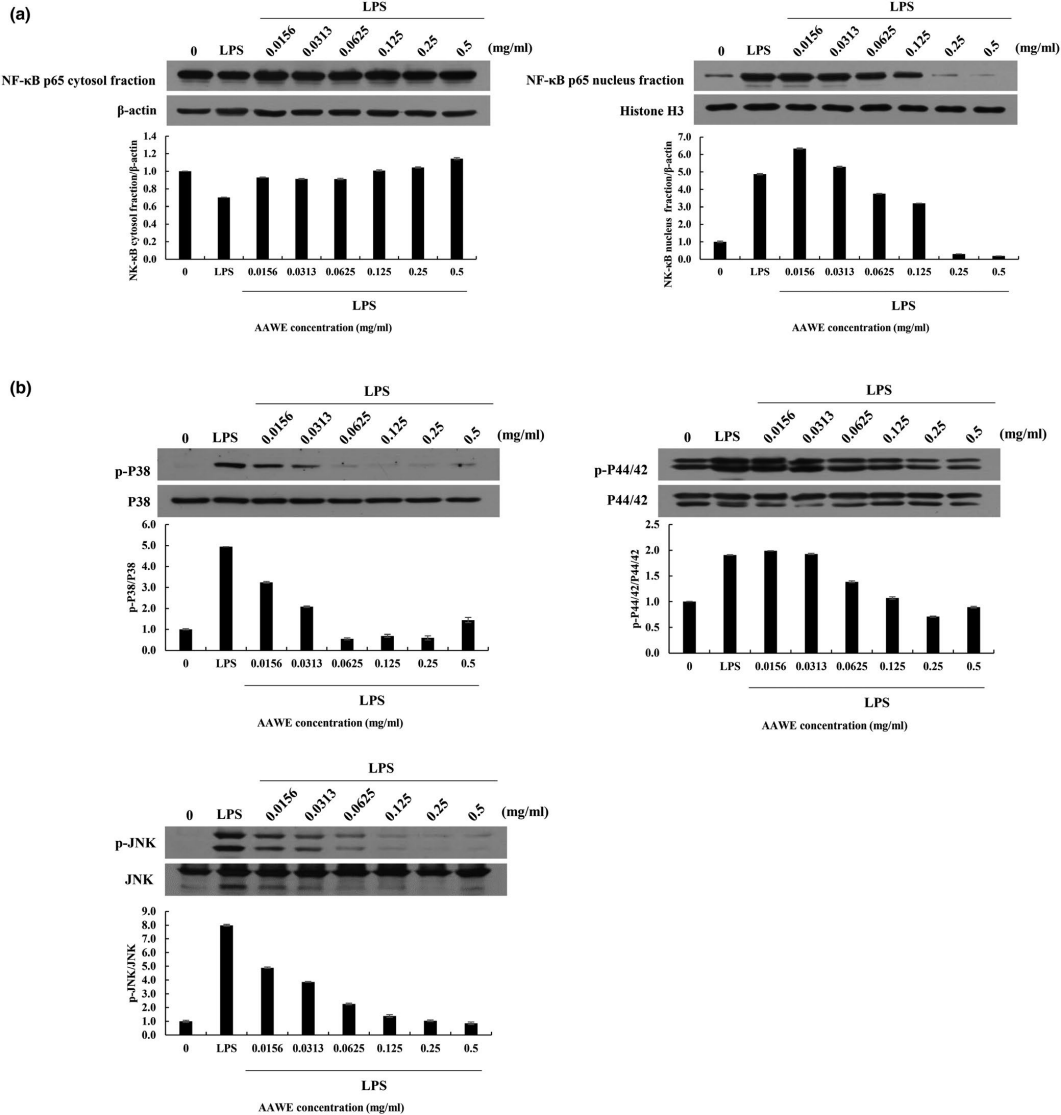
Suppressive effects of *Artemisia annua* water *e*xtract (AAWE) on lipopolysaccharide (LPS)‐induced protein expression of NF‐κB and mitogen‐activated protein kinases (MAPKs) (p38, p44/42, and JNK) in RAW264.7 cells. Raw264.7 cells are grown for 24 hr and then treated with various concentrations of *A. annua* water extract (AAWE) with or without 100 ng/ml LPS for another 24 hr. After protein isolation, Western blot analysis is performed. (a) NF‐κB and (b) MAPK (p38, p44/42, JNK) protein expression is investigated. β‐actin, histone H3, p38, p44/42, and JNK are used as loading controls, and quantification of protein expression is calculated using ImageJ. Blots are representative of three independent experiments. JNK, JUN n‐terminal kinase

In order to investigate the action mechanism of *A. annua* extract that protected against the tissue damage caused by LPS and the subsequent inflammation, we applied AAWE to LPS‐stimulated Raw264.7 cells and examined NF‐κB and MAP kinase signaling (Figure 5a). When LPS stimulation was applied to Raw264.7 cells, the p65 subunit of NF‐κB on the cytoplasmic fraction was not changed, but the p65 on the nuclear fraction increased significantly. Under the same conditions, the pretreatment of AAWE did not change the amount of p65 in the cytoplasm, but the amount of p65 in the nucleus decreased dependent upon the AAWE concentration. This showed that AAWE inhibited the migration of p65 accelerated by LPS into the nucleus and thereby suppressed the gene expression of the inflammatory substances.

### 3.6 AAWE reduced excessive phosphorylation of MAPKs in the LPS‐treated immune cells

ERK1/2 (p44/42), JUN, and p38 MAPKs, as well as NF‐κB, are members of the MAPK family that respond to stimuli in mammals. They are increased in the mouse macrophage, Raw264.7 cells, by LPS stimulation, and they influence the production of inflammatory cytokines. In our study, the total amount of p38, ERK1/2 (p44/42), and JUN proteins was slightly increased in the Raw264.7 cells with 100 ng/ml of LPS (Figure 5b). In contrast to the total amount of MAPKs, phosphorylated ERK1/2 (p44/42), JUN, and p38 were greatly increased with LPS stimulation. Although the total amount of MAPKs was marginally decreased in the presence of AAWE, phosphorylated MAPKs, increased by LPS stimulation, were dramatically decreased with AAWE pretreatment. In particular, the ratio of the phosphorylated form of P38 and JNK versus the total P38 and JNK was lowered, even with <0.1 mg/ml of AAWE.

## 4 DISCUSSION

Lipopolysaccharide (LPS) is a cell membrane component of gram‐negative bacteria, a potent inflammatory agent that promotes natural immune responses in eukaryotes. LPS is composed of hydrophilic saccharide and lipid‐soluble lipid A (Holst, Ulmer, Brade, Flad, & Rietschel, 1996). The lipid A portion binds to the LPS binding protein and CD 14 of the cell membrane, moves to the inner membrane, and then attaches to the LPS receptor complex present in the intracellular membrane. LPS receptor complexes include a variety of CDs and TLRs, such as TLR4, a signaling agent located on the intracellular membrane. These binding units activate various pathways, including NF‐κB, to transmit inflammatory signals throughout the body (Triantafilou & Triantafilou, 2002). In addition to inflammatory responses, LPS is known to cause oxidative stress by increasing the production of free radicals in cells. Excess stimulation of LPS not only secretes proinflammatory mediators such as tumor necrosis factor‐α (TNF‐α), interleukin (IL)‐1, and IL‐6 in macrophages, but also superoxide ( $O_{2}^{-}$ ), hydrogen peroxide (ROS), such as hydroxyl radicals, and excessive activation of NO to induce inducible nitric oxide synthase (iNOS) inflammation, resulting in tissue damage (Mayer & Spitzer, 1993; Neihorster et al., 1992; Streetz et al., 2000).

D‐galactosamine causes damage to liver tissue by altering carbohydrate composition and intracellular calcium ion concentration in the cell membrane through galactose metabolic disorders. Therefore, injection of D‐galactosamine causes liver necrosis due to metabolic disorders, and chronic poisoning leads to cirrhosis and cellular tumors (Francavilla et al., 1986). Since galactosidase, galactose‐1‐furidyl transferase, and uridine diphosphate (UDP)‐glucose are present in large quantities in the liver tissue, the assay system using D‐galactosamine has very high liver specificity and almost no effect on other organs. Thus, D‐galactosamine is used widely to study the functional substances that serve as indicators of liver damage (Maley, Tarentino, McGarrahan, & Delgiacco, 1968). D‐galactosamine‐mediated hepatic injury is caused by the accumulation of UDP hexosamine by galactosamine binding to UDP in cells. It induces the inhibition of RNA and protein synthesis, the abnormal metabolism of carbohydrates, and the degradation of macromolecules (nucleic acid, glycoproteins, glycolipids in membranes, glycogen, etc.), consequently resulting in intracellular organ damage and ultimately leading to hepatocyte necrosis (Kmiec, Smolenski, Zych, & Mysliwski, 2000). Liver failure, as shown in Figure 2, seemed to be mainly due to administration of D‐glaN.

In this study, the hot water extracts of *A. annua* resulted in significant protection against liver damage and improved liver function in the animal model of rapid liver damage caused by LPS and D‐galN. Plant extracts such as dandelion (*Taraxacum officinale*) extract and fischers' ragwort (*Ligularia fischer*) extract have shown anti‐inflammatory and antioxidant activities using similar animal models (Park, Kim, Park, Noh, & Song, 2007; Yu et al., 2015). In addition, natural products such as genistein, quercetin, and chrysophanol have been reported to protect liver damage induced by LPS and D‐galN (Cho et al., 2008; Jiang et al., 2016; Peng et al., 2017). In this study, we confirmed that the effects of *A. annua* extract on improvements in liver damage were made through fundamental signaling systems such as TLR4 inactivation, inhibition of phosphorylation of MAPKs, and blocking the nuclear migration of inflammatory transcription factors.

Lipid A, an LPS component, attaches to LPS binding protein and CD14 at the cell membrane, increasing TLR4 expression as shown in Figure 3, and activated TLR4 transmits the stimulus into the cell. As such, LPS binds to various TLRs, CD11, or CD18, and activates inflammatory signals through NF‐κB, extracellular signal‐regulated kinase 1/2 (ERK1/ 2, p44/ 42 MAP kinase), stress‐activated protein kinase, and p38 MAPKs), and c‐Jun N‐terminal kinase (JNK) pathways (Figure 5). It is known that a single dose of 10 mg of LPS per kg body weight of the mouse evokes an inflammatory response systemically (Hadid, Spinedi, Chautard, Giacomini, & Gaillard, 1999) and a rapid decrease in body weight was shown due to inflammation. This concentration corresponds to the lethal dose (as shown in Table 2) that resulted in the death of 70% (seven of 10) of the experimental animals within 3 hr. Survival was significantly increased in mice when *A. annua* extracts were administered, because the extract suppressed the LPS‐induced systemic inflammatory response and protected against severe liver cell damage. The blood AST and ALT activities, which were increased due to LPS and D‐galN, were significantly decreased in the *A. annua* extract group compared to the vehicle‐treated control group, suggesting improvements in the effect of the extract on liver damage caused by LPS.

NF‐κB is a famous transcription factor initiating an inflammatory response, which promotes the expression of iNOS, thereby increasing the synthesis of inflammatory mediators such as NO (Kooy, Royall, Ischiropoulos, & Beckman, 1994) and increasing the expression of inflammatory precursors such as TNF‐α and IL‐6 (Figures 3 and 4). Increased inflammation subsequently activates the immune responses related to immune cells such as macrophages and neutrophils, resulting in incremental production of ROS and RNS, leading to oxidative stress and promotion of liver lipid peroxidation. Oxidative stress and lipid peroxidation are known to attack biologically effectual proteins and to cause various diseases (Esteve, Ricart, & Fernández‐Real, 2005; Tengku‐Muhammad, Hughes, Cryer, & Ramji, 1996). Injection of LPS and D‐galN into mice caused acute inflammation and liver damage. In this model, administration of *A. annua* extract significantly reduced the early inflammatory cytokines TNF‐α, IL‐6, and IL‐1β (Figure 3) and suppressed acute inflammatory necrosis (Figure 2). The anti‐inflammatory response of the extract was similarly shown in the LPS‐treated macrophage cell line (Figure 4). Inflammation increased the concentration of triglycerides in the blood, which may be caused by (a) incremental release of very low‐density lipoproteins (VLDL) containing triglycerides, or (b) inhibition of lipid protein lipase activity and restriction of triglyceride transfer to subcutaneous fat cells, or (c) increased production of cytokines such as TNF‐α, IL‐1, and IL‐2, IL‐6, and IFN‐α, stimulating VLDL release (Esteve et al., 2005; Tengku‐Muhammad et al., 1996). We have reported that the extract showed excellent antioxidant effects even in a nonalcoholic, high‐fat diet, fatty liver model (manuscript in press).

In most cells, NF‐κB protein dimers (mainly composed of p50 and p65 subunits) remain inactivated in the cytoplasm in combination with IκB (inhibitor κB) proteins. When cells are stimulated externally through pathogen‐associated molecular patterns (PAMPs), TNF receptors (TNFR), TLRs, or IL‐1 receptors (IL‐1Rs), the IKK complex phosphorylates IκB which binds to NF‐κB dimers to inhibit its activation (DiDonato, Hayakawa, Rothwarf, Zandi, & Karin, 1997). Phosphorylated IκB is degraded in the proteasome through a polyubiquitination process, and NF‐κB dimer freed from IκB passes through the nuclear membrane to bind DNA in the nucleus and activate gene transcription (DiDonato et al., 1997). The P65 subunit of NF‐κB protein was moved into the nucleus by LPS stimulation (Figure 5a), and the translocation of p65 into the nucleus was inhibited by the pretreatment of *A. annua* extract. The results suggested that the LPS‐induced activation of NF‐κB p65 could be regulated by *A. annua* extract. Proinflammatory cytokines such as interleukin 1 (IL‐1) and tumor necrosis factor‐α (TNF‐α) activate NF‐κB, and subsequently NF‐κB triggers the expression of other inflammatory genes including cytokines, chemokines, and adhesion molecules. Since inflammation is a complex physiological process, it is hard to define the role of NF‐κB in the inflammatory response in vitro studies only. In this study, we demonstrated that suppression of inflammatory responses of AAWE in LPS/D‐galN‐treated animals demonstrated the similar trend with anti‐inflammatory effect in vitro system based on a mouse macrophage cell line.

Mitogen‐activated protein kinase, consisting mainly of extracellular signal‐regulated kinase (ERK), c‐Jun N‐terminal kinase (JNK/SAPK), and p38 kinase, phosphorylates transcriptional regulators to adjust the expression of various genes involved in cell growth, death, and differentiation. JNK is also called stress‐activated protein kinase (SAPK) and is affected by early inflammatory cytokines such as TNF and interleukin to increase cell death (Kamata et al., 2005). When the activated status of JNK lasts for more than 120 min, apoptosis increases in various cells including hepatocytes (Hanawa et al., 2008). Early inflammatory cytokines such as interleukin and TNF are also known to stimulate p38 signaling (Liu & Min, 2002). In this manner, ROS or early inflammatory cytokines activated the protein kinases, MAP kinase kinase (MAPKK) and MAP kinase kinase kinase (MAPKKK). Subsequently, it phosphorylated MAPKs including JNK, ERK, and p38 kinase (Ho et al., 2004). The excessive phosphorylation of MAPKs was decreased with hot water extract of *A. annua*, especially the reductive tendency of p38 and JNK phosphorylation, which was shown in a dose‐dependent manner.

In summary, the extract of *A. annua* inhibited the early inflammatory response activated by LPS and might prevent cell death and tissue damage by suppressing NF‐kB activation and MAPK activation. We confirmed the efficacy of the extract not only in the mouse macrophage lines but also in the animal models of acute liver injury induced by LPS and D‐galN. Taken together, the *A. annua* extract can be developed as an excellent liver health product to protect liver function and to suppress liver damage.

## CONFLICT OF INTEREST

Chan Young Park, Eunyong Choi, Hee‐Jin Yang, Seong Hyun Ho, Su‐Jin Park, Ki‐Moon Park, and Seon‐Hee Kim declare that they do not have any conflict of interest.

## ETHICAL APPROVAL

All animal experiments were approved by the Animal Research Ethics Committee of KPC (Approval Number: P173010).
